# LncRNA POU3F3 promotes cancer cell migration and invasion in nasopharyngeal carcinoma by up-regulating TGF-β1

**DOI:** 10.1042/BSR20181632

**Published:** 2019-01-25

**Authors:** Wei Li, Xingyu Wu, Wensheng She

**Affiliations:** Department of Otolaryngology, Ezhou Central Hospital, Ezhou City, Hubei Province 436000, P.R. China

**Keywords:** nasopharyngeal carcinoma, lncRNA POU3F3, prognosis, TGF-β1

## Abstract

Up-regulation of lncRNA POU3F3 has been observed in esophageal squamous cell carcinomas, while its expression pattern and functionality in other human disease is unknown. Our study showed that plasma levels of lncRNA POU3F3 and TGF-β1 (transforming growth factor-β) were both increased in nasopharyngeal carcinoma patients than in healthy controls. Plasma levels of lncRNA POU3F3 were not affected by the diameter of primary tumors but increased in patients with tumor metastasis. Plasma levels of lncRNA POU3F3 and TGF-β1 were positively correlated only in nasopharyngeal carcinoma patients but not in healthy controls. Follow-up study showed that high plasma levels of lncRNA POU3F3 were significantly correlated with poor overall survival. LncRNA POU3F3 overexpression and exogenous TGF-β1 treatment led to promoted, while TGF-β1 inhibitor led to inhibited migration and invasion of nasopharyngeal carcinoma cells. TGF-β1 inhibitor partially rescued the inhibited cancer cell migration and invasion caused by lncRNA POU3F3 overexpression. LncRNA POU3F3 overexpression led to down-regulated TGF-β1 expression, while exogenous TGF-β1 and TGF-β1 inhibitor treatment did not significantly change the expression level of lncRNA POU3F3. Therefore, lncRNA POU3F3 may promote cancer cell migration and invasion in nasopharyngeal carcinoma by up-regulating TGF-β1.

## Introduction

As type of malignancy develops from the tissues of nasopharynx, nasopharyngeal carcinoma is a rare cancer in most regions of the world [1]. However, ethnicity significantly affects the occurrence of nasopharyngeal carcinoma [2]. Some populations, such as the populations in southern China, are at high risk for this disease [3]. In China, nasopharyngeal carcinoma affects more than 40,000 new cases and causes more than 20,000 deaths every year [4]. At present, nasopharyngeal carcinoma is considered as one of the major public health problems in China [4]. With efforts made on cancer treatment, long-term survival of nasopharyngeal carcinoma at early stages has been significantly improved [5,6]. However, survival of patients with metastatic nasopharyngeal carcinoma is still poor [5,6].

At present, cancer metastasis treatment and prevention is still a major challenge in clinic [7]. Activation of TGF-β (transforming growth factor-β) signaling participates in cancer metastasis by mediating epithelial-to-mesenchymal transition [8]. In effect, inhibition of TGF-β signaling now is considered as a promising therapeutic target for cancer treatment [9]. A growing body of literature has shown that TGF-β signaling may regulate cancer metastasis through the cross-talk with long non-coding RNAs [10,11], which are also a group of key players in cancer biology [12]. Up-regulation of lncRNA POU3F3 has been observed in esophageal squamous cell carcinomas [13], indicating its oncogenic role, while its expression pattern and functionality in other human disease is unknown. In the present study, we showed that lncRNA POU3F3 promoted cancer cell migration and invasion in nasopharyngeal carcinoma possibly by up-regulating TGF-β1.

## Materials and methods

### Patients and cell lines

Our study included 42 nasopharyngeal carcinoma patients and 38 healthy controls who were admitted by Ezhou Central Hospital from January 2009 to May 2013. Blood was extracted from these participants before treatment to prepare plasma using conventional method. Patients’ inclusion criteria: (1) patients who were diagnosed as nasopharyngeal carcinoma through pathological biopsies; (2) patients without previous history of malignancies and/or family disease history; (3) patients completed treatment and 5-year follow-up. Exclusion criteria: (1) patients who were complicated with other types of malignancies; (2) patients failed to complete treatment or follow-up; (3) patients died of other causes during follow-up. Lymph node metastasis only (LNM) was observed in 12 cases, distant metastasis (DM) was observed in 14 cases, and non-metastasis (NM) was observed in the rest 16 cases. Diameter of the primary tumor was larger than 6 cm in 16 cases and the rest 26 patients had a diameter of the primary tumor smaller than 6 cm. Patient group included 24 males and 18 females, and age ranged from 23 to 68 years with an average age of 45.2 ± 5.1 years. Control group included 20 males and 18 females, and age ranged from 22 to 65 years with an average age of 44.8 ± 4.6 years. These two groups showed similar age and gender distributions. The present study passed the review of Ethics Committee of Ezhou Central Hospital. All participants signed informed consent.

Human nasopharyngeal carcinoma cell lines HTB-43 and C666-1 were purchased from Shanghai Tiancheng Communication Technology Co., Ltd (Shanghai, China). Cells were cultured under conditions recommended by the manufacturer.

### Enzyme-linked immunosorbent assay (ELISA)

TGF-β1 in plasma was detected using Human TGF-β1 ELISA Kit (ab108912, Abcam). All operations were performed following manufacturer’s instructions. Plasma levels of TGF-β1 were normalized to ng/ml.

### RNA extraction and real-time quantitative PCR

Total RNA was extracted from plasma and *in vitro* cultured cells using easy-BLUE™ Total RNA Extraction Kit (iNtRON Biotechnology DR). NanoDrop™ 2000 Spectrophotometers (Thermo Fisher Scientific, U.S.A.) were used to measure RNA concentrations. Reverse transcriptions were used to measure RNA concentrations using Applied Biosystems High-Capacity cDNA Reverse Transcription Kit through following conditions: 25°C for 5 min, 54°C for 20 min, and 75°C for 10 min. All PCR reaction systems were prepared using Luna® Universal qPCR Kit (NEB). Primers of lncRNA POU3F3 and β-actin endogenous control were designed and synthesized by GenePharma (Shanghai, China). Primer sequences were: 5’-AATCACTGCAATTGAAGGAAAAA-3’ (forward) and 5’-CCTTGTTTTCCAACCCTTAGACT-3’ (reverse) for lncRNA POU3F3; 5’-AAATCTGGCACCACACCTTC-3’ and 5’-GGGGTGTTGAAGGTCTCAAA-3’ (reverse) for β-actin. PCR reactions were performed through following conditions: 1 min at 95°C, then 15 s at 95°C, and 35 s at 59°C for 40 cycles. PCR products were sequenced to make sure that correct products were obtained. Data normalizations were performed using 2^−ΔΔ^*^C^*_T_ method.

### Cell transfection

Vectors expressing lncRNA POU3F3 were designed and prepared by GenePharma (Shanghai, China). All cell transfections were performed using Lipofectamine 2000 Reagent (11668-019, Invitrogen, Carlsbad, U.S.A.). All operations were performed following manufacturer’s instructions. Cells only treated with lipofectamine 2000 reagent were control cells. Cells transfected with empty vectors were negative control cells.

### Cell migration and invasion assay

At 24 h after transfection, expression of lncRNA POU3F3 was detected by real-time quantitative PCR (RT-qPCR) and overexpression rate above 200% was achieved. Cell migration and invasion abilities were measured using Cell Migration and Invasion Assay Kit (BD Biosciences, U.S.A.). Cell suspensions at a cell density of 3 × 10^4^ cells were prepared using plasma-free medium. Cells were cultivated in the upper chamber with 0.1 ml cell suspension in each well, and the lower chamber was filled with cell culture medium containing 20% FBS. Cells were cultivated for 24 h. After that, the upper chamber was collected and cleaned, followed by 0.5% Crystal Violet (Sigma-Aldrich, U.S.A.) staining at room temperature for 10 min. Stained cells were counted under an optical microscope. Upper chamber was pre-coated with Matrigel (356234, Millipore, U.S.A.) before invasion assay.

### Total protein extraction and Western blot

ReadyPrep™ Protein Extraction Kit (Bio-Rad) was used to extract total protein and Pierce BCA Protein Assay Kit (Thermo Fisher Scientific) was used to measure protein concentrations. After denaturing, electrophoresis was performed using 10% SDS-PAGE gel. Following gel transfer to PVDF membranes, membranes were blocked in 5% fat-free milk for 2 h at room temperature. Primary antibodies used in Western blot included rabbit anti-human TGF-β1 (1:1300, ab92486, Abcam) and rabbit anti-human GAPDH antibody (1:1000, ab9485, Abcam). Secondary antibody was goat anti-rabbit IgG-HRP (1:1200, MBS435036, MyBioSource). Western blot was performed using conventional method. ECL (Sigma-Aldrich, U.S.A.) was used to develop signals. Data normalizations were performed using Image J v1.46 software.

### Statistical analysis

All experiments were performed three times and mean ± standard deviation was calculated. Statistical analyses were performed using SPSS19.0 (SPSS Inc., U.S.A.) software. Correlations between expression levels of lncRNA POU3F3 and TGF-β1 were analyzed using Pearson Correlation Coefficient. Patients were divided into high (*n*=17) and low (*n*=25) lncRNA POU3F3 expression groups according to Youden index. Survival curves of both groups were plotted using Kaplan–Meier method and compared by Log-rank (Mantel-Cox) test. Student’s *t*-test was used for comparisons between two groups. One way analysis of variance followed by Tukey test was used for comparisons among multiple groups.* P*<0.05 was considered to be statistically significant.

## Results

### Plasma levels of lncRNA POU3F3 and TGF-β1 were increased in nasopharyngeal carcinoma patients

**Figure 1:**
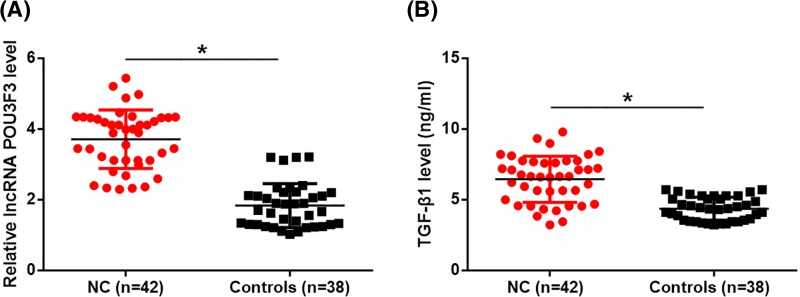
Plasma levels of lncRNA POU3F3 and TGF-β1 were increased in nasopharyngeal carcinoma patients Plasma levels of lncRNA POU3F3 and TGF-β1 in nasopharyngeal carcinoma patients and healthy controls were measured by RT-qPCR and ELISA, respectively. Compared with healthy controls, plasma levels of lncRNA POU3F3 (**A**) and TGF-β1 (**B**) were increased in nasopharyngeal carcinoma (NC) patients (*P<0.05).

RT-qPCR results showed that plasma levels of lncRNA POU3F3 were increased in nasopharyngeal carcinoma (NC) patients (Figure 1A, *P*<0.05). ELISA results showed that plasma TGF-β1 was also significantly up-regulated in NC patients than in healthy controls (Figure 1B, *P*<0.05)

### Plasma levels of lncRNA POU3F3 were affected tumor metastasis

**Figure 2:**
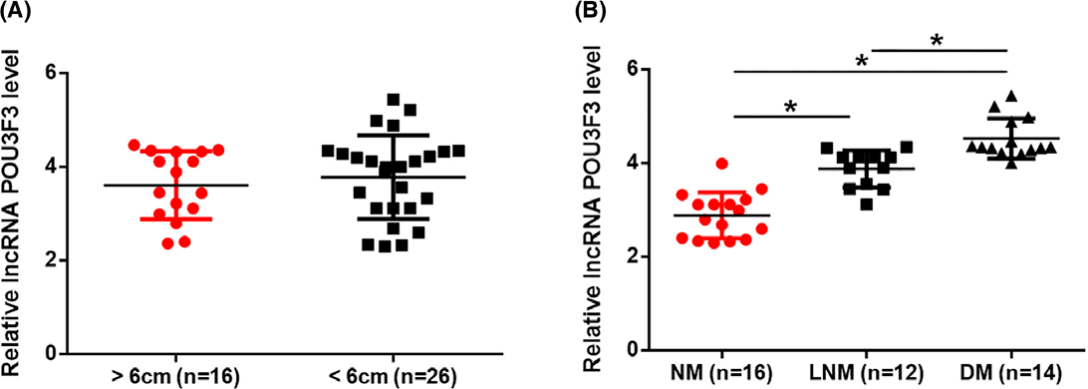
Plasma levels of lncRNA POU3F3 were affected tumor metastasis Patients were divided into different groups according to primary tumor diameter and the existing of tumor metastasis. Plasma levels of lncRNA POU3F3 were compared among groups. Plasma levels of lncRNA POU3F3 were not affected by tumor size (**A**) but were affected tumor metastasis (**B**) (*P<0.05).

Among the 42 patients, diameter of the primary tumor was larger than 6 cm in 16 cases and the rest patients had a diameter of the primary tumor smaller than 6 cm. As shown in Figure 2A, plasma levels of lncRNA POU3F3 were not affected by tumor size. Among these patients, LNM was observed in 12 cases, DM was observed in 14 cases and NM was observed in the rest 16 cases. As showed in Figure 2B, plasma levels of lncRNA POU3F3 were significantly higher in LNM group than in NM group (*P*<0.05). In addition, plasma levels of lncRNA POU3F3 were also significantly higher in DM group than in LNM group. In addition, plasma levels of lncRNA POU3F3 increased slightly with the increase of clinical stages, while no significant differences were found (data not shown).

### Plasma levels of lncRNA POU3F3 and TGF-β1 were positively correlated only in nasopharyngeal carcinoma patients

**Figure 3:**
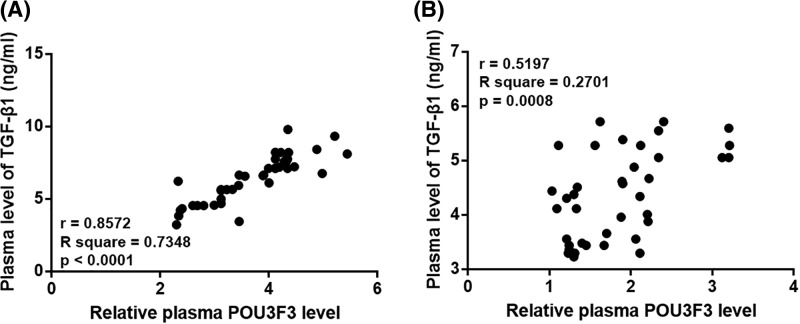
Plasma levels of lncRNA POU3F3 and TGF-β1 were positively correlated only in nasopharyngeal carcinoma patients Pearson Correlation Coefficient showed that plasma levels of lncRNA POU3F3 and TGF-β1 were positively correlated in nasopharyngeal carcinoma patients (**A**) but not in healthy controls (**B**).

Correlations between plasma levels of lncRNA POU3F3 and TGF-β1 were analyzed using Pearson Correlation Coefficient. A significantly positive correlation between plasma levels of lncRNA POU3F3 and TGF-β1 was observed in nasopharyngeal carcinoma patients (Figure 3A). In contrast, no significant correlation between plasma levels of lncRNA POU3F3 and TGF-β1 was found in healthy controls (Figure 3B).

### High plasma levels of lncRNA POU3F3 were significantly correlated with poor overall survival

**Figure 4:**
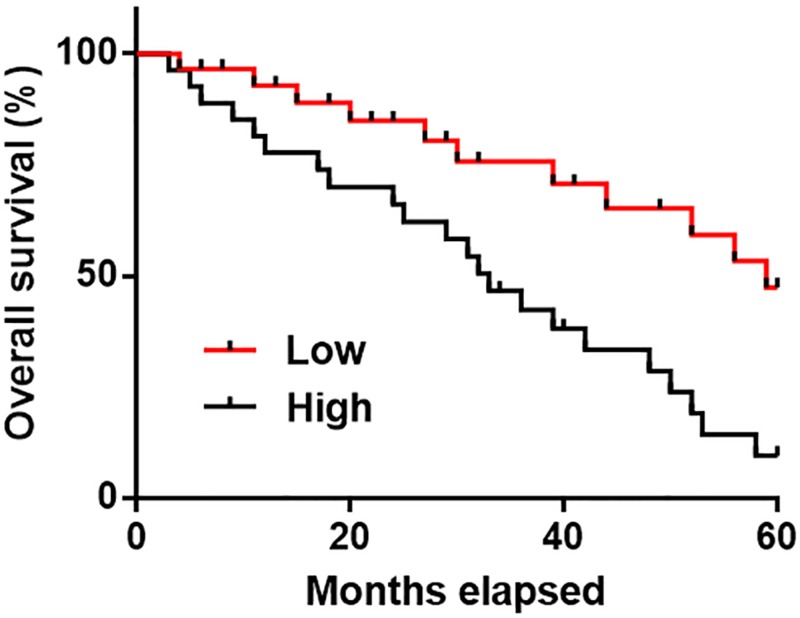
High plasma levels of lncRNA POU3F3 were significantly correlated with poor overall survival Survival curve analysis showed that patients with high plasma levels of lncRNA POU3F3 had shorter survival time compared with patients with low plasma levels of lncRNA POU3F3.

Patients were followed up for 5 years or until their deaths. Patients were divided into high (*n*=17) and low (*n*=25) lncRNA POU3F3 expression groups according to Youden index. Survival curves of both groups were plotted using Kaplan–Meier method and compared by Log-rank (Mantel-Cox) test. As shown in Figure 4, patients with high plasma levels of lncRNA POU3F3 showed significantly worse survival compared with patients with low plasma levels of lncRNA POU3F3 (*P*=0.0026).

### LncRNA POU3F3 overexpression led to up-regulated TGF-β1 expression in cells nasopharyngeal carcinoma cell lines HTB-43 and C666-1

**Figure 5:**
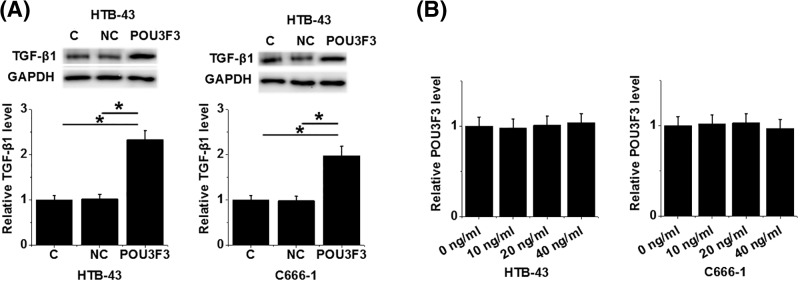
LncRNA POU3F3 overexpression led to up-regulated TGF-β1 expression in cells nasopharyngeal carcinoma cell lines HTB-43 and C666-1 TGF-β1 expression was detected by Western blot after lncRNA POU3F3 expression vector transfection. Up-regulated expression of TGF-β1 was observed in cells of both HTB-43 and C666-1 cell lines after lncRNA POU3F3 overexpression (**A**), while RT-qPCR results showed no significant changes in expression levels of lncRNA POU3F3 after treatment with exogenous TGF-β1 (**B**) (*P<0.05).

After transfection, expression of lncRNA POU3F3 and TGF-β1 was detected by RT-qPCR and ELISA, respectively. Compared with control (C) and negative control (NC), up-regulated expression of TGF-β1 was observed in cells of both cell lines after lncRNA POU3F3 overexpression (Figure 5A, *P*<0.05). However, no significant changes in expression levels of lncRNA POU3F3 were observed after treatment with exogenous TGF-β1 (Sigma-Aldrich, St. Louis, MO, U.S.A.) at doses of 10, 20 and 40 ng/ml for 24 h (Figure 5B).

### LncRNA POU3F3 overexpression promoted nasopharyngeal carcinoma cell migration and invasion through TGF-β1

**Figure 6:**
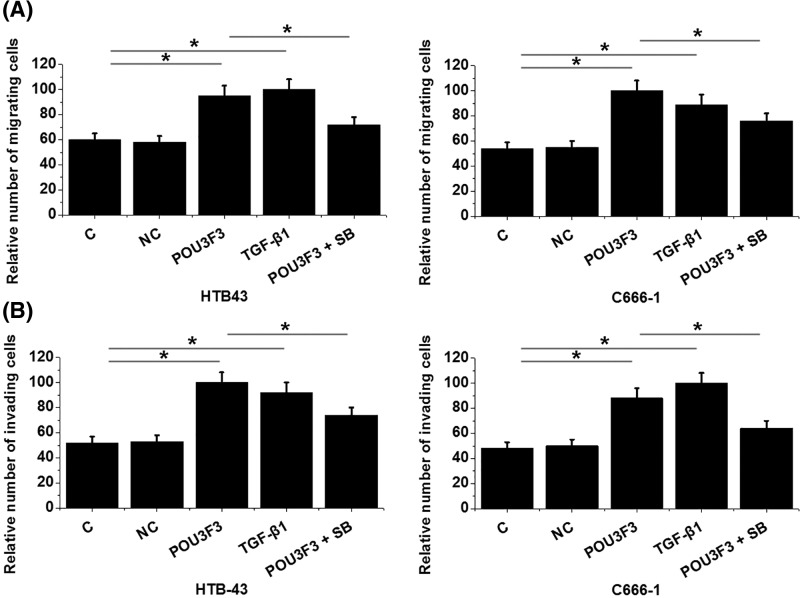
LncRNA POU3F3 overexpression promoted nasopharyngeal carcinoma cell migration and invasion through TGF-β1 Cell migration and invasion abilities were tested by Transwell migration and invasion assays. LncRNA POU3F3 overexpression and TGF-β1 (10 ng/ml) treatment led to significantly promoted cancer cell migration (**A**) and invasion (**B**). In addition, treatment with TGF-β inhibitor SB431542 (SB, 10 nM, Sigma-Aldrich) attenuated the enhancing effect of lncRNA POU3F3 overexpression on cell migration and invasion (*P<0.05).

After transfection, cell migration and invasion abilities were measured through cell migration and invasion assay. Compared with control (C) and negative control (NC), lncRNA POU3F3 overexpression and TGF-β1 (10 ng/ml) treatment led to significantly promoted cancer cell migration (Figure 6A) and invasion (Figure 6B) (*P*<0.05). In addition, treatment with TGF-β inhibitor SB431542 (SB, 10 nM, Sigma-Aldrich) attenuated the enhancing effect of lncRNA POU3F3 overexpression on cell migration and invasion (*P*<0.05).

## Discussion

A recent study has shown that POU3F3 as a novel lncRNA is up-regulated in plasma of patients with esophageal squamous cell carcinoma, and the altered plasma lncRNA POU3F3 showed diagnostic potentials for this disease [13]. However, the functions of lncRNA POU3F3 in esophageal squamous cell carcinoma, as well as its involvement in other human diseases are unknown. In the present study, we showed that lncRNA POU3F3 was also up-regulated in plasma of nasopharyngeal carcinoma patients. Up-regulation of plasma lncRNA POU3F3 led to promoted nasopharyngeal carcinoma cell migration and invasion possibly by up-regulating TGF-β1.

The development of nasopharyngeal carcinoma induces altered expression of a large set of lncRNAs in human genome [14]. Numerous previous studies have shown that differentially expressed lncRNAs participate in nasopharyngeal carcinoma by promoting or inhibiting cancer development [15,16]. However, reports on a specific involvement of an lncRNA in a particular step of cancer progression are rare. It is known that lncRNA POU3F3 is up-regulated in plasma of patients with esophageal squamous cell carcinoma [13]. In the present study, we showed that plasma levels of lncRNA POU3F3 were also increased in patients with nasopharyngeal carcinoma. In addition, plasma levels of lncRNA POU3F3 were affected by tumor metastasis but not by metastasis. Our *in vitro* experiments also showed that lncRNA POU3F3 overexpression promoted the migration and invasion of nasopharyngeal carcinoma cells. Those data suggest the specific involvement of lncRNA POU3F3 in the metastasis of nasopharyngeal carcinoma.

Interestingly, our study observed a significantly positive correlation between plasma levels of lncRNA POU3F3 and TGF-β1 in patients with nasopharyngeal carcinoma. TGF-β as a key player in cancer biology promotes tumor metastasis by inducing epithelial-to-mesenchymal transition [17]. It is known that TGF-β can promote cancer progression by up-regulating the expression of certain lncRNAs [18]. In addition, activation of TGF-β signaling during cancer development can also be regulated by upstream lncRNAs [19]. Our *in vitro* cell experiments suggest that lncRNA POU3F3 is likely an upstream activator of TGF-β1 in the regulation of migration and invasion of cancer cells. These data provided new insights to the molecular mechanism of the pathogenesis of nasopharyngeal carcinoma.

Our study failed to elucidate the mechanism of the regulatory role of lncRNA POU3F3 in the expression of TGF-β1, which is a direction of our future studies. However, the interaction between lncRNA POU3F3 and TGF-β1 is likely mediated by certain pathological factors due to the lack of significant correlation between lncRNA POU3F3 and TGF-β1 in healthy controls. Our follow-up study revealed that high plasma level of lncRNA POU3F3 may serve as a biomarker for the poor prognosis of nasopharyngeal carcinoma, which may provide guidance for clinical studies.

In conclusion, lncRNA POU3F3 was up-regulated in nasopharyngeal carcinoma. LncRNA POU3F3 may participate in tumor metastasis but not growth by interacting with TGF-β1.

## Abbreviations

DM: distant metastasis
LNM: lymph node metastasis
NM: non-metastasis
RT-qPCR: real-time quantitative PCR

## References

[1] Jemal A., Bray F., Center M.M. (2011) Global cancer statistics. CA Cancer J. Clin. 61, 69–90 10.3322/caac.20107 21296855

[2] Hamilton S.N., Ho C., Laskin J. (2016) Asian versus non-Asian outcomes in nasopharyngeal carcinoma: a North American population-based analysis. Am. J. Clin. Oncol. 39, 575–580 10.1097/COC.0000000000000091 24879476

[3] Cao S.M., Simons M.J. and Qian C.N. (2011) The prevalence and prevention of nasopharyngeal carcinoma in China. Chin. J. Cancer 30, 114–119 10.5732/cjc.010.10377 21272443 PMC4013340

[4] Wei K.R., Zheng R.S., Zhang S.W. (2014) Nasopharyngeal carcinoma incidence and mortality in China in 2010. Chin. J. Cancer 33, 381–387 25096544 10.5732/cjc.014.10086 PMC4135367

[5] Sun X., Su S., Chen C. (2014) Long-term outcomes of intensity-modulated radiotherapy for 868 patients with nasopharyngeal carcinoma: an analysis of survival and treatment toxicities. Radiother. Oncol. 110, 398–403 10.1016/j.radonc.2013.10.020 24231245

[6] Su S.F., Han F., Zhao C. (2012) Long-term outcomes of early-stage nasopharyngeal carcinoma patients treated with intensity-modulated radiotherapy alone. Int. J. Radiat. Oncol. Biol. Phys. 82, 327–333 10.1016/j.ijrobp.2010.09.011 21035959

[7] Khan N. and Mukhtar H. (2010) Cancer and metastasis: prevention and treatment by green tea. Cancer Metastasis Rev. 29, 435–445 10.1007/s10555-010-9236-1 20714789 PMC3142888

[8] Katsuno Y., Lamouille S. and Derynck R. (2013) TGF-β signaling and epithelial–mesenchymal transition in cancer progression. Curr. Opin. Oncol. 25, 76–84 10.1097/CCO.0b013e32835b6371 23197193

[9] Connolly E.C., Freimuth J. and Akhurst R.J. (2012) Complexities of TGF-β targeted cancer therapy. Int. J. Biol. Sci. 8, 964–978 10.7150/ijbs.4564 22811618 PMC3399319

[10] Pádua Alves C., Fonseca A.S., Muys B.R. (2013) Brief report: the lincRNA Hotair is required for epithelial‐to‐mesenchymal transition and stemness maintenance of cancer cell lines. Stem Cells 31, 2827–2832 10.1002/stem.1547 24022994

[11] Yuan J., Yang F., Wang F. (2014) A long noncoding RNA activated by TGF-β promotes the invasion-metastasis cascade in hepatocellular carcinoma. Cancer Cell 25, 666–681 10.1016/j.ccr.2014.03.010 24768205

[12] Gutschner T. and Diederichs S. (2012) The hallmarks of cancer: a long non-coding RNA point of view. RNA Biol. 9, 703–719 10.4161/rna.20481 22664915 PMC3495743

[13] Tong Y.S., Wang X.W., Zhou X.L. (2015) Identification of the long non-coding RNA POU3F3 in plasma as a novel biomarker for diagnosis of esophageal squamous cell carcinoma. Mol. Cancer 14, 3 10.1186/1476-4598-14-3 25608466 PMC4631113

[14] Yang Q.Q. and Deng Y.F. (2015) Genome‐wide analysis of long non‐coding RNA in primary nasopharyngeal carcinoma by microarray. Histopathology 66, 1022–1030 10.1111/his.12616 25406670

[15] Bo H., Gong Z., Zhang W. (2015) Upregulated long non-coding RNA AFAP1-AS1 expression is associated with progression and poor prognosis of nasopharyngeal carcinoma. Oncotarget 6, 20404–20418 10.18632/oncotarget.4057 26246469 PMC4653014

[16] Gong Z., Zhang S., Zeng Z. (2014) LOC401317, a p53-regulated long non-coding RNA, inhibits cell proliferation and induces apoptosis in the nasopharyngeal carcinoma cell line HNE2. PLoS One 9, e110674 10.1371/journal.pone.0110674 25422887 PMC4244030

[17] Massagué J. (2008) TGFβ in cancer. Cell 134, 215–230 10.1016/j.cell.2008.07.001 18662538 PMC3512574

[18] Fan Y., Shen B., Tan M. (2014) TGF-β–induced upregulation of malat1 promotes bladder cancer metastasis by associating with suz12. Clin. Cancer Res. 20, 1531–1541 10.1158/1078-0432.CCR-13-1455 24449823

[19] Mondal T., Subhash S., Vaid R. (2015) MEG3 long noncoding RNA regulates the TGF-β pathway genes through formation of RNA–DNA triplex structures. Nat. Commun. 6, 7743 10.1038/ncomms8743 26205790 PMC4525211

